# Initiatives for promoting educator wellbeing: a Delphi study

**DOI:** 10.1186/s40359-024-01724-7

**Published:** 2024-04-22

**Authors:** Patrick P, Reupert A, Berger E, Morris Z, Diamond Z, Hammer M, Hine R, Fathers C

**Affiliations:** 1 School of Educational Psychology and Counselling, Faculty of Education, Monash University, 19 Ancora Imparo, VIC 3800 Clayton, Australia; 2 Monash Rural Health, Monash University, Warragul, Australia; 3 School of Education, Culture & Society, Faculty of Education, Monash University, Clayton, Australia; 4 BeyondBlue, Melbourne, Australia

**Keywords:** Educator wellbeing; wellbeing initiatives; Delphi technique, Collegial relationships, Leadership, Intervention design

## Abstract

**Background:**

Growing demands on educators have resulted in increased levels of burnout and stress and decreasing wellbeing. This study aimed to establish expert consensus on the key characteristics required in prospective educator wellbeing initiatives.

**Methods:**

The Delphi approach is a process of forecasting that is based on the aggregated opinion of panel members (or experts) within a field of study. Using a Delphi approach, academic and practitioner expertise were sought over a two-rounds, with 17 and 14 participants in each round respectively. The study aimed to identify how systemic factors (e.g., leadership) could be utilised to promote educator wellbeing. The study also sought expert consensus on enablers and barriers for engagement in educator wellbeing initiatives.

**Results:**

Findings highlighted the importance of fostering positive relationships with colleagues, communities and families, and the active role of wellbeing teams to promote wellbeing initiatives. The need for leaders to address their own wellbeing and build trust within teams was also identified. Panel members identified the need for prospective funding to prioritise wellbeing initiatives. There was also a preference for ongoing initiatives rather than stand-alone wellbeing events that conveyed the ongoing importance of managing one’s wellbeing.

**Conclusions:**

This paper presents practical recommendations that can be used to inform the development and evaluation of future initiatives and policy. Applying the consensus derived from this study is likely to make wellbeing initiatives more viable and facilitate uptake amongst educators.

## Introduction

Educator wellbeing is associated with teaching ability and performance [1, 2], and subsequently impacts children’s wellbeing [2] and learning [3]. However, there is a paucity of evidence-based programs designed to promote educator wellbeing [4]. In Australia, individuals employed in the early childhood sector are commonly referred to as ‘educators’ whilst those who teach in primary and secondary schools are typically referred to as ‘teachers’. For consistency, in this paper, we adopt the term ‘educator’ to encompass both early childhood educators as well as teachers employed in primary and secondary schools. Extensive research documents high stress levels and depression [5], emotional exhaustion [6] and low levels of mental health in educators to be common factors leading to high attrition rates. Scholarly statistics and the Australian media claim that between 30 and 50% of educator attrition occurs in the first five years [7]. Attrition has found to be a significant concern amongst male educators and is even more concerning when recruitment of male educators into the industry remains low [8]. Additionally, alienation and stereotyping by their students were factors that led to increased negative emotions on the job for secondary school educators [9].

Although there are governance, policy, and workplace differences between early childhood settings, primary and secondary schools, there are also similarities in their workplaces, including the relational nature of their role, goals of practice, and expert knowledge about learning. Their settings also share high levels of workplace stress [10, 11], intense emotional demands [1, 12], and continuously evolving policy and practice frameworks [10, 11]. Thus, it is critical to identify the most effective means of promoting wellbeing for these educator groups.

### Educators and stress

Compared to the general population, primary and secondary educators report higher levels of stress and depression [13] and report lower wellbeing compared to other professional occupations [14]. In the USA, 46% of 7200 surveyed educators reported feeling stressed daily [15], and in the UK, a 2017 survey found 81% of educators had considered leaving the profession in the past 12 months due to excessive workload [16]. In Australia, educators make more mental health claims than any other professional group [17]. Early childhood educators have reported similar, deleterious outcomes. For example, one study found that 63% of 533 family childcare providers experienced job-related stress [18], and another reported that across various settings, early childhood educators’ depressive symptoms ranged from 6 to 24% [19]. A review of preschool educator wellbeing found low wages in particular impacted staff turnover and job satisfaction, with other stressors including time and administrative demands [20]. A recent report revealed that an estimated 46.8% of Australian educators are considering leaving the profession with the next 12 months [21]. Adding to this concern, 2023 witnessed a 20% decline in enrolment numbers in education degree programs, resulting in the Federal Government stating that Australia was facing an “unprecedented teacher supply and retention challenges” [22]. Taken together, the lack of adequate resources and increasing workload demands continue to make educators vulnerable to emotional exhaustion and burnout [23].

For all educator groups, job-related stressors may include managing children’s sometimes challenging behaviour, conflict with families [24], problematic relationships with colleagues, limited resources, administrative demands, and time pressures [20, 25–28]. The emotional involvement at the core of teaching and caring for children and young people [29] can be another source of stress, especially if the educator feels unable to respond to a child’s emotional, social, or behavioural needs [4].

In the occupational literature, high job-related stress levels are related to lower productivity, and greater levels of sickness, absenteeism, and presenteeism [30]. Specific to teaching, educator stress impacts their capacity to interact and respond to children’s needs [31], and where repeated patterns of student misbehaviour can perpetuate stress, increasing the likelihood of punitive or reactive responses [32]. Early childhood educators experiencing depression are found to be less sensitive and more withdrawn in their interactions with children [33]. Stress is one of the causes for 30% of early childhood providers in the USA leaving the profession [34] and is one of the reasons that between 19 and 30% of new primary and secondary educators leave the field within the first five years [35].

### Education leaders and wellbeing

School and early childhood leaders have their own wellbeing needs. One Australian wide survey, comprising 2,248 school leaders showed that nearly 97% of principals worked overtime and reported high levels of burnout, stress, and depressive symptoms [36]. Simultaneously, education leaders are known to play a critical role in promoting a positive, safe, and productive working environment. Research has found that specific leadership behaviours were associated with educator wellbeing, including having clearly defined objectives and tasks, and the ability to communicate their vision and mission [37]. Educator wellbeing is heavily influenced by effective school leadership [38], and accordingly is a key component in promoting occupational wellbeing in educational contexts [39]. Similarly, in their interviews with Israeli educators, results indicated that school principals play an essential role in promoting educators’ wellbeing by creating a positive emotional climate, promoting positive collegial relationships, and demonstrating genuine concern for educators [40]. Similarly, research highlights the role of early childhood directors in providing emotional support and developing an inspiring workplace environment [41].

### Educator wellbeing

Job related stress refers to an individual’s reaction to threats emulating from their workplace [42], while job related wellbeing has been operationalised in terms of job satisfaction, stimulation, and enthusiasm [43]. Educator wellbeing is important as it has been linked to effective teaching practices, educator efficacy, collegial relationships, and student achievement [43, 44]. Moreover, educators with higher reported rates of wellbeing employ adaptive coping strategies [45, 46], report job satisfaction, and show organisational commitment [47]. Conversely, a lack of educator wellbeing has been associated with emotional exhaustion and reduced personal satisfaction that can result in indifferent or negative attitudes toward students [28].

A focus on wellbeing assumes that “positive functioning is not simply surviving stress; it also entails thriving physically, mentally, socially, and professionally” [47]. Other scholars have operationalised wellbeing as a multifaceted construct that involves attaining a state of equilibrium where an individual possesses the psychological, social, and physical resources required to manage the psychological, social, and physical challenges they encounter [48]. Similarly, for educators, wellbeing is informed by individual (e.g., a positive attitude or a healthy work-life balance), relational (e.g., the quality of the educators’ relationships with their peers), and contextual (e.g., working climate and policy initiatives) factors, underscoring the multifaceted nature of wellbeing [49]. Accordingly, the current study adopts a holistic definition of wellbeing as follows [50]:

…dynamic state, involving the interaction of individual, relational, work-environmental and socio-political aspects and contexts. Educators’ well-being is the responsibility of the individual *and* the agents of these contexts, requiring ongoing direct and indirect supports, across psychological, physiological and ethical dimensions” (p. 276).

**Fig. 1:**
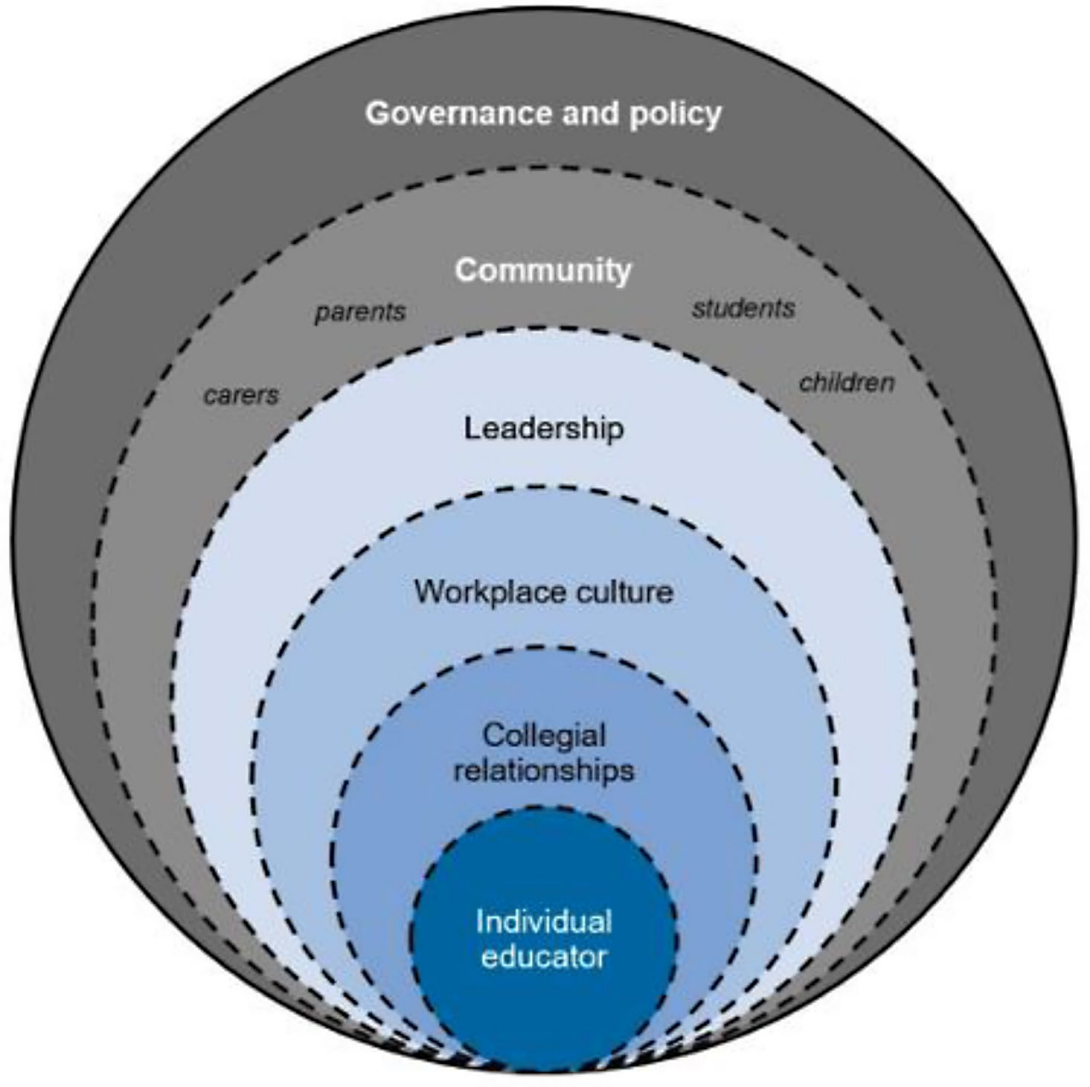
Educator wellbeing: an ecological framework

The definition is extended further by adopting the ecological framework to conceptualize educator wellbeing [51]. The framework in particular highlights the significance of workplace on educators’ wellbeing and is featured using two separate nested circles labelled ‘workplace culture’ and ‘leadership’. Additionally, the broken lines as depicted in Fig. 1 emphasise the interrelated and permeable nature of these domains on educator wellbeing [51].

### Educator wellbeing interventions

Traditionally, intervention programs targeting educator wellbeing have focused on ameliorating educator stress, rather than promoting wellbeing [47]. Such interventions deliver training to educators to manage the adverse physiological and psychological effects of stress, with a focus, for example, on mindfulness, adaptive coping, or yoga [52–54]. Previous research has also reported on the efficacy of mindfulness for educators as a means of developing adaptive coping and mitigating their stressful experiences [52]. Such interventions, however, do not attend to the environments and conditions in which educators work, and accordingly, largely fail to consider the multifaceted nature of wellbeing. The WISE study conducted in the UK with 1,722 educators adds further evidence of the need to account for broader factors, such as school culture in promoting educator wellbeing [55]. The WISE study measured educators’ wellbeing and secondary outcomes including depression, absence, student well-being and mental health difficulties. The intervention comprised of mental health first aid training for educators, a mental health awareness session and a confidential staff peer support service. Following two years of data collection, results revealed no difference in mental health and wellbeing in educators and students in the intervention group compared to the control group. The authors highlighted the need for future interventions to consider targeting systemic factors, such as school culture and level of perceived support, and posit that these might have a stronger influence on promoting educator wellbeing, and in turn, promote student wellbeing outcomes.

The contention to focus on systemic factors was also reinforced in a recent review of educator wellbeing programs [4]. The review identified 19 evidence-based educator wellbeing programs worldwide, with only one study evaluating the effectiveness of a program on early childhood educator wellbeing and the majority focusing on individual determinants of wellbeing, rather than broad systemic issues. It has been argued that a focus on individual components of educator wellbeing is inappropriate because when “strategies for promoting wellbeing are individualistic rather than collective, efforts to promote educator wellbeing become precarious and unsustainable” [11]. Likewise, interviews with senior early childhood managers about workplace wellbeing underscored a need for initiatives to address structural factors (e.g., health and safety, operational challenges, enhancing collegial relationships [41]. To date, an ecological approach to educator well-being that considers structural determinants of educator wellbeing is not well understood nor appropriately operationalised, which makes it difficult to develop holistic educator wellbeing interventions or initiatives [56]. This study aims to identify expert consensus driven priorities for promoting wellbeing initiatives for educators. The results of this study can be used to inform the development of future educator wellbeing initiatives.

### Study design and aims

A Delphi study was conducted to establish expert consensus on the key characteristics of effective educator wellbeing initiatives. The aims of the Delphi study were four-fold: (i) obtain consensus on the focus of wellbeing initiatives, (ii) ascertain the role of early childhood and school leaders in promoting wellbeing, (iii) identify who is responsible for wellbeing initiatives, and finally (iv) identify factors that might hinder or promote educator wellbeing initiatives.

## Method

The Delphi process involves engaging with content experts over sequential questionnaires (or rounds) until consensus among the experts is reached [57]. By using anonymised and sequential questionnaires, the Delphi method minimises domination by powerful individuals in a group [58] and promotes the expression of novel ideas and feedback [59, 60]. With each successive round, experts are given an opportunity to adjust their initial responses after reviewing the anonymous, collated responses of other experts, generated from previous survey rounds. Within the current study, two rounds were undertaken. Experts were derived from three categories: (i) researchers with at least five years of research experience in the field of educator wellbeing, (ii) practitioners with at least five years of experience developing and/or delivering educator wellbeing initiatives, or (iii) individuals who fit both criterions. The study was a funded project conducted by researchers from (blinded for review purposes) University, in collaboration with an Australian mental health wellbeing support organisation. The project was approved by the Monash University Human Research Ethics Committee (Project ID: 27,308). Data collection occurred between February and March 2021 and the study materials (i.e., questionnaires and data file) are available upon request.

### Recruitment procedures

Purposive sampling was employed to identify a panel of informed experts who had knowledge of educator wellbeing. A systematic review on educator wellbeing was used to identify experts with research experience in educator wellbeing. A separate Google search was conducted to identify individuals from Australian government agencies, not-for-profit organisations, and social media interest groups that promoted educator wellbeing. From this search, 12 educational centres and organisations across Australia were contacted about the study. Further to this, 15 Australian and 11 international education experts were contacted respectively about this study. These individuals were also requested to disseminate the study to their professional networks, resulting in a snowball effort to recruit academics and practitioners, with a specific interest in educator wellbeing. Additionally, the professional networks of the research team that comprised education and educational psychology researchers, were used to identify relevant participants.

### Study design: round one

A mixture of open-ended and rating questions was employed in round one. Open-ended questions were used to generate a broad range of responses (e.g., the focus of educator wellbeing initiatives). Conversely, rating questions were employed to ascertain questions relating to preference. For example, experts were asked to rate which initiative modality (e.g., synchronous, asynchronous, or hybrid delivery of programs) they perceived would be most suitable in targeting educators. Questions were designed based on key themes and issues in educator wellbeing identified in the literature. Specifically, a systematic review of educator wellbeing initiatives was conducted prior to this study, which revealed that while there were numerous educator initiatives, many of these were targeted at the individual educator [4]. Additionally, with wellbeing being a multifaceted construct, the current study sought to delve more deeply into systemic factors (e.g., the role of education leaders) relating to educator wellbeing. Questions relating to program design, modality and engagement were included as a way of informing practical considerations.

### Study design: round two

Questions in round two were drawn from responses collated in the first round and consisted of a series of rating and ranking questions. Questions in round two required experts to rate the preferred modality of wellbeing initiatives, delivery format (e.g., online, face-to-face, or hybrid initiatives), and rank topics in order of their importance. A copy of the questionnaire used in round two is available from the authors.

### Participants’ demographics

A total of 17 and 14 participants completed the survey in rounds one and two respectively. The age range of participants in round one was between 30 and 63 years (*M* = 44.1, *SD* = 10.5). Experts in round two were aged between 30 and 65 (*M* = 49.0, *SD* = 12.5). In rounds one and two, most experts identified as researchers accounting for 58.8% and 50.0% respectively. Table 1 details the gender, professional background, field, and nationality of all participants.

**Table 1:** Summary of participant demographics for rounds one and two

		Round 1			Round 2		
Demographic category		Researcher n = 10	Practitioner n = 5	Researcher and practitioner n = 2	Researcher n = 7	Practitioner n = 4	Researcher and practitioner n = 3
Gender	Woman	9	5	0	6	3	3
	Man	1	0	2	1	0	0
	Non-binary/gender diverse	0	0	0			
	Prefer not to say	0	0	0	0	1	0
Age range		30–58	31–63	42–62	30–56	32–64	53–65
Professional background	Education	8	2	1	3	2	2
	Psychology	4	3	1	3	1	1
	Public health	0	0	0	1	0	0
	Aboriginal educator	0	0	0	0	1	0
Nationality	Australian	9	4	1	5	3	2
	American	1	0	0	1	0	0
	British	0	0	1	1	0	0
	Singaporean	0	1	0	0	1	0
	Greek	0	0	0	0	0	1

### Data analysis

Data for rounds one and two were collected using the Qualtrics online survey package. Responses were screened and cleaned in a Microsoft Excel spreadsheet for analysis. In round one, six phases of thematic analysis were used to analyse the open-ended questions [61]. The first two authors engaged in an iterative process to derive themes from responses obtained in round one. Each author coded the raw data set independently. Following which, different segments of responses were picked at random, and codes were compared between each author. In round two, a consensus threshold was determined based on previous Delphi studies pertaining to mental health [62]. As questions in round two comprised rating and ranking questions, descriptive analysis, such as median and mean ranks were calculated, using IBM SPSS Statistics Version 20, to determine if consensus was attained. Missing data were dealt with by computing central tendencies based on the total number of participants who provided a response to that item, to ensure results were not skewed. Consensus for rating questions was reached when at least 70% of participants’ responses fell between two points above and below the mean, as supported by others [63]. Consensus for ranking questions was ascertained by calculating mean ranks using SPSS Statistics Version 27.

### Round one results

#### Educator wellbeing foci

Participants were asked an open-ended question about what focus was important for promoting educator wellbeing for early childhood, primary, and secondary school educators. Four themes were identified out of a total of 30 responses. The themes and respective frequency of citations are denoted in superscript: (i) developmental-specific knowledge relating to student needs^11^, (ii) knowledge and skills in caring for oneself ^8^, (iii) bolstering educator identity and purpose^7^, and (iv) fostering supportive relationships^4^. The following section uses verbatim responses from participants to facilitate the expansion of themes.

A need for age-specific developmental knowledge was considered important. For early childhood educators, emphasis was placed on understanding age-appropriate milestones. In primary school settings, there was an emphasis on equipping educators with skills to have age-appropriate conversations about potentially challenging topics (e.g., climate change, political upheaval), whilst an understanding of hormonal changes and working with adolescence as they shift from dependence to independence was identified for secondary educators. Experts also agreed on the importance of imparting educators with skills and resources to help them care for themselves. The theme included aspects such as being aware of burnout signs and learning to self-regulate, increasing emotional understanding of self and setting boundaries. The third theme was around bolstering educators’ identity and purpose where experts identified the role of wellbeing initiatives in facilitating positive *“teacher identity development… maintain(ing) passion and purpose” [Researcher (age 30) in pre-service and secondary education].* The fourth and final theme on fostering supportive relationships emphasised *“the importance of social support at school and outside of work”*, community connections, and building skills to develop and maintain collegial relationships with colleagues, and early childhood and/or school leadership *[Researcher (age 41) in pre-service, primary, and secondary education].*

### Delivery of wellbeing initiatives

Although social media platforms and online resources met consensus, face-to-face delivery remained the most preferred option. Table 2 shows the top three options that met consensus threshold.

**Table 2:** Preferred mediums for educator wellbeing initiatives (*N* = 17)

Preferred medium	Number of participants who provided endorsement	Mean (%)
Option 1: Face-to-face Professional Development	16	8.56 (94.1)
Option 2: Social media platforms (e.g., YouTube, Facebook, Instagram, Blogs)	12	7.40 (70.6)
Option 3: Online resources (e.g., brochures, pamphlets, tip sheets shared via online platforms)	12	6.88 (70.6)

*Note* Top three responses were most popular. Note Other options provided on survey include: websites, smartphone apps, emails, mainstream media outlets, and print media

In relation to delivery format, participants were asked to rate between synchronous (live or in real time), asynchronous (not in real time and self-paced), and hybrid (both synchronous and asynchronous) initiatives from 0 (*not useful*) to 10 *(very useful*). Although all options met consensus threshold, hybrid initiatives were the most preferred delivery format, receiving 88.2% of agreement (Table 3).

**Table 3:** Preferred delivery formats for educator wellbeing initiatives (*N* = 17)

Preferred delivery format	Number of participants who provided endorsement	Mean (%)
Option 1: Hybrid initiatives (i.e., combination of real-time online activities and self-paced, activities)	15	8.31 (88.2)
Option 2: Asynchronous (not in real time)	14	7.00 (82.4)
Option 3: Synchronous (in real time activities)	12	7.25 (70.6)

Other pedagogical matters when delivering wellbeing initiatives were identified and from the nine options provided, four met the consensus threshold (Table 4), with mentoring and scenario-based learning rated as the most useful.

**Table 4:** Preferred professional development pedagogies when delivering educator wellbeing initiatives (*N* = 17)

Preferred medium	Number of participants who provided endorsement	Mean (%)
Option 1: Mentoring initiatives	16	8.35 (94.1)
Option 2: Scenario-based learning and case studies	16	8.29 (94.1)
Option 3: Opportunities for reflective practice	14	7.25 (82.4)
Option 4: Self-directed learning	12	5.25 (70.6)

*Note* Top four responses were most popular. Note Other options provided on survey include: quizzes, videos, infographics, and opportunities to engage in groupwork

### Responsibility of promoting wellbeing initiatives

Participants were asked to identify those who they thought should be responsible for promoting educator wellbeing initiatives. A total of 20 responses were received, which were groups into four categories: (i) school or early childhood wellbeing teams^10^, (ii) school principals and/or early childhood leadership and/or senior educators^4^, (iii) guests from external organisations^3^ and governing agencies, such as Department of Education^3^.

### The role of leadership in promoting educator wellbeing

Participants were asked to describe the role of early childhood and school leaders in promoting educator wellbeing. Responses predominantly related to upskilling education leaders to cultivate a healthy workplace culture^9^ that values collegial and respectful relationships as well as leaders’ capacity to care and communicate^7^. The following quotes captures the essence of each theme respectively:

*“the importance of environment - in terms of collegial support, importance of positive leadership which reinforces positive interrelationships and rewards staff, need to have respectful relationships (no bullying etc), a workable workload, support for each other to promote morale - even if it’s a stressful school/centre - the feeling that they are all in it together.” [Researcher (age 56) in primary and secondary education].*

*“Leaders should demonstrate that they care for their staff, they need to listen to the opinions/perspectives of staff. Trust/care is foundational. Without that, nothing else will work.” [Researcher (age 41) in pre-service, primary and secondary education].*

Several participants emphasised the need for education leaders to be aware of, and address their own wellbeing, explained as follows:

*“The importance of prioritising their own wellbeing (leaders) and the impact that can have on staff and kids (students).” [Practitioner (age 50) in early childhood and primary education].*

### Promoting engagement

Experts were asked to identify ways to encourage educator engagement and participation in wellbeing initiatives. Fourteen responses were obtained and further grouped into three broad categories: (i) included leadership support in prioritising wellbeing initiatives^6^ (e.g., through inclusion in key performance indicators), (ii) flexi-mode delivery and interactive learning materials^4^ and (iii) providing supervision and mentoring opportunities^2^. Other responses obtained to a lesser frequency included the need for more funding and having external speakers present on various topics.

### Barriers to, and strategies for improving, engagement in educator wellbeing initiatives

Participants were asked to identify common barriers that might prevent educators from engaging in wellbeing initiatives, along with strategies they thought might overcome these barriers. Five key barriers included: (i) unsupportive workplace cultures, (ii) lack of leadership, (iii) lack of time, (iv) inadequate infrastructure (e.g., funding, technological devices), and (v) lack of interest in and commitment to wellbeing initiatives.

The identified strategies to overcome identified barriers were largely structural in nature and involved promoting healthy workplace cultures. Strategies included adopting whole-of-school approaches, making educator wellbeing initiatives mandatory and requiring schools/early childhood settings to report on educator wellbeing as Key Performance Indicators (KPIs). Additionally, normalising help-seeking amongst educators, allocating protected time for educators to engage in wellbeing initiatives, providing flexible, manageable, self-paced activities, and the provision of technology and funding were regarded as useful strategies for promoting engagement in wellbeing initiatives. Conducting periodic school or centre wide surveys to assess educators’ needs, interests and/or gaps in knowledge, and inviting guest speakers and external experts were also identified.

### Round two results

#### Educator wellbeing foci

As outlined in Table 5, the only focus that reached consensus among participants in round two was ‘fostering supportive relationships with others’ (i.e., colleagues, community, and families), with 10 out of 14 participants ranking it within their top three ranks.

**Table 5:** Frequency and mean ranks for educator wellbeing initiative focus (*N* = 14)

Topic areas	Mean rank	Number of participants who ranked it as top 3
Fostering supportive relationships with others including colleagues, community and families	1.75	10
Classroom-specific skills	3.00	6
Strengths, agency and purpose passion	3.75	8
Learning adaptive coping skills	4.00	6
Learning ways to achieve work-life balance	4.00	4
Education on self-care and wellbeing	4.50	8

*Note* Shaded areas in the table are reflective of items that did not reach consensus threshold

### Delivery

In regard to the duration, as reflected in Table 6, consensus was achieved for ongoing initiatives and hybrid initiatives, which were ranked in first and second place respectively.

**Table 6:** Mean rank and overall ranking for preferred duration (*N* = 14)

Topic areas	Mean rank	Overall ranking
Ongoing or recurring initiatives	1.64	1
Hybrid initiatives (i.e., dependent on school and early childhood calendar)	1.86	2
Short, modular courses	2.50	3

### Responsibility of promoting wellbeing initiatives

As shown in Table 7, school and early childhood wellbeing teams and external guest speakers were ranked as the most preferred personnel to deliver wellbeing initiatives.

**Table 7:** Frequency and mean ranks for promotion of wellbeing initiatives (*N* = 14)

Topic areas	Mean rank	Overall ranking	Number of participants who ranked it as top 3
School and/or early childhood wellbeing teams	1.38	1	12
Delivered by guest speakers or experts in various topics	2.46	2	11
By senior educators	2.83	-	9
By school leadership	3.33	-	6

*Note* Shaded areas in the table are reflective of items that did not reach consensus threshold

### The role of leadership in promoting educator wellbeing

Participants were asked to rank items developed in round one, in relation to the role of leaders in wellbeing initiatives. Ways to become better communicators and to build trust within teams was ranked at first place, followed by skills to build a collegial workplace culture (e.g., promoting positive interrelationships and rewards for staff, building in time with educators’ schedule to encourage collaboration) and last, understanding how their wellbeing impacts organisational culture and staff wellbeing (Table 8).

**Table 8:** Frequency and mean ranks in relation to the role of school and early childhood leaders (*N* = 14)

Topic areas	Mean rank	Overall ranking	Number of participants who ranked it as top 3
Learning ways of becoming better communicators and building trust within teams	2.35	1	11
Skills to build a collegial workplace culture	2.58	2	10
Understand the impact of leadership wellbeing on the school community	2.96	3	11

*Note* Ranks were placed on a scale from 1 = most important to 3 = least important

### Engagement

With retention of participation in initiatives being an identified barrier in round one, experts were asked to rank engagement strategies. Out of eight options, six met the consensus threshold of requiring at least 70% or more of participants to rate the option three or higher on a 4-point Likert scale, with a mean rating score of 3.25 or higher (see Table 9).

**Table 9:** Effectiveness of engagement strategies to retain educators in wellbeing initiatives (*N* = 14)

Topic areas	Mean rating	Overall ranking	Number of participants who ranked 3 or more (on a 4-point Likert scale)
Provision of funding and financial support for wellbeing initiatives	3.46	1	12
Interactive resources	3.42	2	13
Flexi-mode delivery options	3.35	3	13
Provision of supervision and mentorship opportunities	3.35		12
Support of school leadership	3.35		11
Options to customize PD or to mix-and-match training depending on educators’ interest and area of need	3.28	4	12
Engaging experts	2.92	-	11
Prioritising wellbeing initiatives	2.92	-	10

*Note* Means are rated on scale of 1 = very ineffective to 4 = very effective. *Note* Shaded areas in the table are reflective of items that did not reach consensus threshold

### Barriers to engagement

Participants were asked to rank common barriers to educator participation in wellbeing initiatives, derived from the thematic analysis of round one data. Ratings were provided from 1 ‘most important to address’ to 5 ‘least important to address’. Table 10 presents the mean ranks for all barriers. The only barrier that reached consensus was ‘absence or perceived lack of leadership support’. Although lack of time and a culture of not prioritising mental wellbeing were the next closest options that served as barriers, these narrowly missed the consensus threshold.

**Table 10:** Frequency and mean ranks on barriers to engaging educators (*N* = 14)

Topic areas	Mean rank	Overall rank	Number of participants who ranked it as top 3 (out of 14)
Absence or perceived lack of leadership support	2.31	1	11
Culture of not prioritising mental wellbeing	2.58	-	9
Lack of time	2.62	-	9
Inadequate infrastructure (e.g., provision of technology to improve educators’ accessibility to initiatives)	3.42	-	8
Lack of interest and/or wellbeing initiatives perceived as extra work	4.08	-	4

*Note* Ranks were placed on a scale of 1 = most important to address, 5 = least important to address. *Note* Shaded areas in the table are reflective of items that did not reach consensus threshold

### Strategies to overcome barriers

In round two, participants were asked to rate the strategies that were derived in round one, in terms of their effectiveness to overcome barriers (Table 11). Themes were identified from free-text responses in round one, with a total of 13 strategies identified. Round two participants were asked to rate each strategy of its level of effectiveness, from 1 ‘*very ineffective*’ to 4 ‘*very effective*’.

**Table 11:** Effectiveness of proposed strategies to overcome barriers to educator wellbeing (*N* = 14)

Topic areas	Mean rating	Overall rank	Number of participants who ranked it as top 3
Whole-of-school approaches that include school and early childhood leaders championing wellbeing practices in school	3.64	1	13
Allocate protected time for educators to engage in wellbeing initiatives	3.64	-	13
Normalising help-seeking amongst educators	3.42	2	13
Target early childhood and school leaders’ wellbeing as a first step	3.42	-	12
Access to more funding for educators to access professional development activities	3.35	3	14
Self-paced professional development activities on wellbeing topics	3.30	4	13

*Note* Means are rated on scale of 1 = very ineffective to 4 = very effective

## Discussion

From a two-round Delphi survey, this study sought to generate consensus regarding educator wellbeing initiative foci, delivery of initiatives, the role of leadership, and the enablers and barriers for educators when engaging in wellbeing initiatives. Findings highlight specific and practical ways to develop and promote wellbeing initiatives for educators in early childhood, primary, and secondary settings.

### Wellbeing focus and pedagogies

According to experts, the ability to foster supportive and collegial relationships amongst educators, in the community and with families was the most essential focus for educator wellbeing initiatives. Recent studies have highlighted the importance of connectedness and trust amongst staff for promoting educator wellbeing and overall work fulfilment [64, 65], and to mitigate the emotional demands and exhaustion associating with the educator role [66]. Similarly, the importance of solidarity and collegial support amongst early childhood educators for promoting wellbeing, especially during COVID-19 related lockdowns, were emphasised as being vital to overcoming work-related challenges [67]. Consensus from experts in this study was to focus on educators’ relational climate within and outside of their work settings, which suggests a need for more emphasis to be given to social capital. Social capital is a relational construct that by its nature is dependent on the interaction of individuals [68]. Furthermore, the growing focus on social capital in promoting educator wellbeing fundamentally stems from education being a highly relational industry [69]. Current findings on collegial relationships corroborate with previous research that has identified high-quality interactions between educators as a pivotal source of advice, information, peer support and learning that are pre-requisites in creating positive work environments [70].

Experts also agreed that ongoing initiatives to promote wellbeing were preferred over stand-alone initiatives. A preference emerged for face-to-face or a hybrid model (e.g., real-time workshop combined with self-paced online activities) of wellbeing initiatives. A recent study involving 10 early career educators found that in person trainings proved more valuable than online modalities as it allowed for interaction and learning from other educators [71]. Similarly, in a sample of Australian early childhood educators, a fundamental need for continual, reflective practice emerged as being pivotal in addressing educators’ psychological needs [72].

### Responsibility of promoting wellbeing initiatives

Consensus was achieved for school wellbeing team and experts from external organisations to be responsible for wellbeing initiatives. The school wellbeing team was seen as appropriate as it was comprised of individuals who were responsible to manage and advocate for wellbeing needs while remaining neutral. Wellbeing teams within schools have the added advantage of understanding a school’s internal systems and operations, and thus in a better position to provide tailored wellbeing initiatives. External service providers were regarded as having evidence-based insights on the current practices of corporate wellbeing and were also deemed suitable providers of wellbeing initiatives for educators. Of note, consensus was not achieved for leaders to assume this role in the current study. However, in some instances school leaders were seen as an appropriate choice, if they were considered to be role-models of wellbeing practices themselves [73]. Similarly, focus group findings from an earlier phase of this research revealed that educators valued leaders who prioritised wellbeing in themselves and throughout the school community. Being visible and serving as role-models in self-care enabled educators to see their school leaders in a different light, which had positive impacts on their wellbeing. It is well documented, however, that school leaders face high levels of stress as their roles become increasingly complex [74, 75]. Taken together, these findings suggest that to have maximum impact, educational leaders may want to consider how they implement wellbeing practices in their own lives, and consequently the examples they might be setting for their school community.

### The role of leaders in wellbeing initiatives

Experts agreed that leaders need to be upskilled in effective communication, building trust within teams, and be imparted with strategies to promote a collegial workplace culture. Previous studies have also emphasised the role that educational leaders play in facilitating staff collaboration, having open and honest communication with staff, and building on communication skills such as active listening, to enhance wellbeing and the quality of interactions between educators and leaders [76–78]. Experts also agreed that leaders’ capacities for leading and supporting others is intimately associated with their own level of self-awareness, self-management, and regulation [79]. The experts in this study have extended previous findings by highlighting the importance of both intrapersonal and interpersonal dimensions of wellbeing for leaders and where leaders need to be aware not only of their own emotions but of the relationship between their own emotions and the impact this has on others in the school environment.

### Barriers to engagement

An absence or perceived lack of leadership support was rated most strongly as being the barrier of educator engagement in wellbeing initiatives. Likewise, emotional support from the principal (e.g., through positive regard, comfort, and understanding) was reported by a group of special and general educators to be the most important form of social support received in their role [80]. School and early childhood leaders are the “gatekeepers” of innovations that occur in their settings [81] and their leadership is critical in creating a vision around wellbeing for staff and students. Nonetheless, their ability to support educator wellbeing largely depends on their own mental health and wellbeing, making it a key priority [22].

### Strategies to overcome barriers

A whole-school approach achieved consensus amongst experts as being pivotal in overcoming the barrier of the perceived lack of support from educational leadership. A whole-school approach aims to integrate skill development into daily interactions and practices using collaborative efforts that include all staff, educators, families, and children [83]. Extant research has shown initiatives at the whole-school level yield the most successful outcomes [83, 84] as they attempt to implement changes that are embedded into daily practice and school culture, involve all members of staff, support parental engagement, and coordination with external agencies [85]. Apart from adopting a whole-school approach, consensus was also obtained for practical strategies, such as, protected time for educators to engage in wellbeing initiatives. A UK study found that educators reported high wellbeing when they were provided with high quality (i.e., meaningful) workloads, educator autonomy, and flexible work arrangements [64]. High quality workloads are defined as those which have a clear and direct benefit to pupils [64]. Compared to high volume and low-quality workloads, it was suggested that schools review educators’ marking policies in addition to finding ways to enhance high-quality workloads [64]. In the current study, participants also called for a need to prioritise early childhood and school leaders’ wellbeing as a first step towards promoting a wider culture of wellbeing, a finding which resonates with the other findings [82].

## Conclusion and implications

This study specifically focused on gathering the views of educator wellbeing from academics and industry experts. Findings emphasised the importance of facilitating collegial relationships with colleagues and the community, and through leaders’ capacity to promote open communication and building trust within teams. This study highlights the important and influential role that education leaders play in organisational culture and wellbeing outcomes for educators. Additionally, the salience of collegial relationships that permeated across this study emphasises the highly relational nature of education. Similarly, collegial and relational aspects were salient themes that emerged in a focus group study with Australian educators [51]. Bolstering educator identity and a sense of purpose also holds important practical implications in attracting and retaining educators in the profession. Education policy that places emphasis on quality workloads (i.e., tasks that have clear benefits to students), offers a degree of autonomy on lesson planning, allocated time for collaboration and collegial partnerships to be built, as well as streamlined assessment and marking procedures are some strategies to consider, to foster educator identity and purpose [64].

## Limitations

Notwithstanding the contributions, several limitations should be acknowledged. As is common in Delphi methodology, results are based on the opinions and experiences of the participants and other samples of participants may result in different priorities [86, 87]. Furthermore, the current study did not record participants’ racial or ethnic backgrounds, which may have had some bearing on participants’ views on wellbeing and serves as an avenue for future research to explore. The small sample size particularly in round two is a limitation. However, other studies have considered a sample size of 12 or more to be adequate for Delphi studies [88]. The modality of employing sequential questionnaire sent via email may be a further limitation as it did not offer a platform for interactive or real-time participant discussion. Although electronic modalities are advantageous in expanding access to experts, it could have posed some barriers. For example, participants may have felt isolated or unsure of how to answer questions that may have resulted in brevity in response, which might otherwise have been expanded upon if they had been prompted by others. Lastly, it needs to be acknowledged that while there are commonalities between early childhood, primary and secondary educators, there are also nuances between these education sectors. In particular, the population and developmental needs of students across these sectors are likely to vary considerably and present with different challenges and needs for educators. Hence, future research may benefit from conducting a separate Delphi study with specific groups of educators (e.g., early career educators, early childhood educators) to gain a more nuanced understanding of the challenges and needs that different settings and student populations might pose on educators.

Whilst this study underscores the need for systemic wellbeing initiatives and policies, much of the available literature to date, remain on individual-level wellbeing initiatives [4]. Hence, ongoing efforts are also needed to evaluate systemic wellbeing policies on the subjective wellbeing of educators. Additionally, given the ever-evolving field of education, particularly since the Covid-19 pandemic that poses new challenges [89], there is a need for periodic research capturing educators’ voices, to ensure policies and practices remain in tandem to the needs of educators.

It is widely acknowledged that teaching is stressful, with the profession as a whole witnessing increased burnout and attrition rates [90, 91]. These findings provide relevant and timely insights on the ‘what’ and ‘how’ wellbeing initiatives should prioritise, to bolster educator wellbeing in early childhood and school settings. The emphasis on building relatedness, fostering trust in teams, and having leadership that champion wellbeing initiatives are some of the key practical suggestions identified in the current study. The findings from this study will be useful as a foundation for designing, developing, and piloting new wellbeing initiatives for educators.
